# Cuts Both Ways: Proteases Modulate Virulence of Enterohemorrhagic *Escherichia coli*

**DOI:** 10.1128/mBio.00115-19

**Published:** 2019-02-26

**Authors:** Lauren D. Palmer, Eric P. Skaar

**Affiliations:** a Department of Pathology, Microbiology, and Immunology and the Vanderbilt Institute for Infection, Immunology and Inflammation, Vanderbilt University Medical Center, Nashville, Tennessee, USA

**Keywords:** EHEC, commensal, microbiota, protease, virulence

## Abstract

Enterohemorrhagic Escherichia coli (EHEC) is a major cause of foodborne gastrointestinal illness. EHEC uses a specialized type III secretion system (T3SS) to form attaching and effacing lesions in the colonic epithelium and outcompete commensal gut microbiota to cause disease. A recent report in *mBio* (E. A. Cameron, M. M. Curtis, A. Kumar, G. M. Dunny, et al., mBio 9:e02204-18, 2018, https://doi.org/10.1128/mBio.02204-18) describes a new role for gut commensals in potentiating disease caused by EHEC. Proteases produced by EHEC and the prevalent human commensal Bacteroides thetaiotaomicron cleave proteins in the EHEC T3SS translocon that modulate T3SS function. B. thetaiotaomicron protease activity promotes translocation of bacterial effectors required for lesion formation. These results describe a new role for the microbiota in gastrointestinal disease that could uncover future treatments to prevent the spread of gastroenteritis.

## Abstract (precis)

Enterohemorrhagic Escherichia coli (EHEC) is a major cause of foodborne gastrointestinal illness. EHEC uses a specialized type III secretion system (T3SS) to form attaching and effacing lesions in the colonic epithelium and outcompete commensal gut microbiota to cause disease.

## COMMENTARY

Enteric bacterial pathogens intimately interact with gut commensals during the course of infection. Generally, the gut microbiota confers resistance to enteric pathogens, primarily through competition for resources and training of mucosal immunity (1). Many Gram-negative enteric pathogens use specialized translocation systems such as the type III secretion system (T3SS) to modulate the host environment and overcome competition from the resident microbiota. This group includes attaching and effacing (A/E) pathogens such as enterohemorrhagic Escherichia coli (EHEC). EHEC is responsible for a significant amount of foodborne illness and causes severe gastroenteritis, enterocolitis, and bloody diarrhea. The Centers for Disease Control and Prevention (CDC) estimates that Shiga toxin-producing E. coli, including the oft-publicized EHEC serotype O157:H7, cause approximately 265,000 infections each year in the United States. Bovine products are the most common source of EHEC foodborne illness. Cows are a major natural reservoir for EHEC, where colonization by this organism is largely asymptomatic despite the formation of similar A/E lesions as in humans.

EHEC and other A/E pathogens contain a locus of enterocyte effacement (LEE) that encodes a T3SS that enables intimate attachment to the colonic epithelium, inducing colonic crypt hyperplasia and generation of a characteristic actin “pedestal.” The EHEC T3SS translocon includes the needle sheath protein EspA and the pore complex proteins EspB and EspD. EspABD facilitate insertion of the bacterial translocated intimin receptor (Tir) into the host membrane and translocation of EHEC effectors into the cytoplasm of host colonocytes. Tir then interacts with bacterial intimin to mediate A/E.

**FIG 1:**
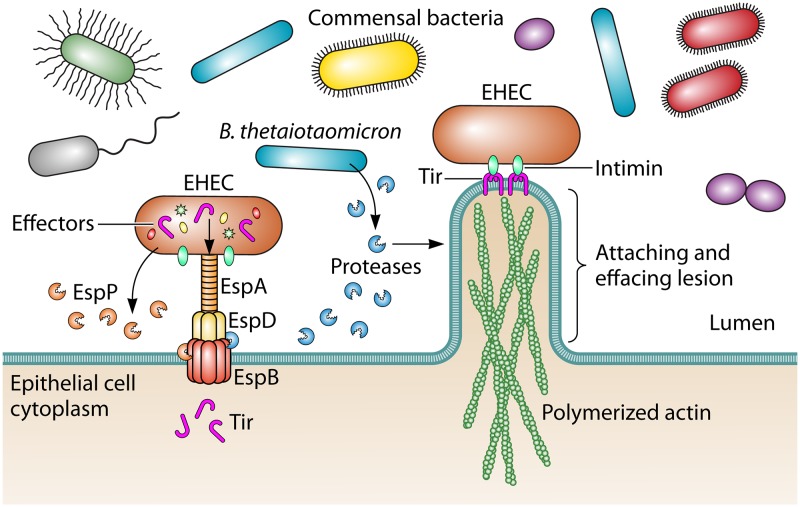
Enterohemorrhagic Escherichia coli interacts with the colonic epithelium through the type III secretion system (T3SS). The T3SS translocon includes the needle sheath protein EspA and pore proteins EspB and EspD. The translocon injects effector proteins into the host cytoplasm required for formation of the attaching and effacing lesion, including the translocated intimin receptor Tir. The EHEC protease EspP cleaves EspB, inhibiting this process. In contrast, the gut commensal B. thetaiotaomicron produces as-yet-unidentified proteases that cleave EspB and promote effector translocation and lesion formation.

This intimate association allows A/E pathogens to outcompete commensal microbiota in the colon, as the T3SS is dispensable in germfree mice (2). In the intestinal lumen, the commensal microbiota effectively outcompetes A/E pathogens for shared carbohydrate substrates (2). Formation of A/E lesions induces oxygenation of the mucosal surface, allowing aerobic respiration by A/E pathogens and an accelerated pathogen growth rate above anaerobic commensal bacteria (3). However, we are beginning to appreciate that these interactions are incredibly complex, and recent research has revealed that the human gut commensal Bacteroides thetaiotaomicron promotes virulence of A/E pathogens (4), suggesting that certain microbiota-pathogen interactions may facilitate, rather than prevent, disease. In a recent study published in *mBio*, Cameron et al. demonstrate that EHEC and B. thetaiotaomicron proteases differentially impact EHEC pathogenesis (5). While an EHEC protease inhibits EHEC virulence, B. thetaiotaomicron proteases promote EHEC T3SS maturation and A/E lesion formation (Fig. 1).

To better understand how B. thetaiotaomicron modulates EHEC pathogenesis, Cameron et al. tested the effect of B. thetaiotaomicron on the expression, maintenance, and function of EHEC T3SS components. Coculture of EHEC with B. thetaiotaomicron promoted transcription of *espABD*, as previously described. However, by analyzing the secreted EHEC proteome by mass spectrometry and immunoblotting, Cameron et al. discovered that growth of EHEC with B. thetaiotaomicron led to reduced abundance of EspB, EspA, and EspD at various time points. At earlier time points, multiple EspB cleavage products were observed. Subsequent analysis determined that secreted serine proteases from both EHEC and B. thetaiotaomicron contributed to EspB cleavage. Specifically, the EHEC protease EspP led to cleavage of the 37-kDa EspB to a 30-kDa product. Purified EspP was also found to cleave EspA and EspD. Incubation with B. thetaiotaomicron led to EspB cleavage to 34- and 36-kDa products. The authors were unable to identify the responsible B. thetaiotaomicron protease despite genetic disruption of seven putatively secreted proteases, including one identified in the supernatants of coculture with EHEC. However, B. thetaiotaomicron encodes 35 putative secreted proteases, suggesting functional redundancy in protease activity may be responsible for EspB cleavage.

Cameron et al. then determined the cleavage sites of the 30- and 34-kDa fragments using Edman degradation amino acid sequencing. This analysis determined that EspP cleaves EspB between Ala80 and Val81, while the unidentified B. thetaiotaomicron protease(s) cleaves EspB between Leu31 and Ser32 in the N terminus. The authors showed that disruption of these sites by amino acid substitution protected EspB from cleavage by the respective proteases. Both of these cleavage sites are in the N-terminal region that is likely extracellular in related strains (6), suggesting these sites are accessible to secreted proteases in the gut. Interestingly, while the B. thetaiotaomicron cleavage site is in a region known to be tolerant to mutation, the EspP cleavage site is immediately downstream of a region important for EspD interaction and identified as essential for function (6).

Cameron et al. next probed the role of translocon cleavage on T3SS function in a human epithelial cell model of A/E lesions, or pedestals. As previously reported, coculturing with B. thetaiotaomicron significantly increased EHEC pedestal formation (4). In addition, deletion of *espP* when EHEC is cocultured with B. thetaiotaomicron significantly increased pedestal formation above coculturing with wild-type EHEC. To measure translocation of the effector Tir, a Tir–β-lactamase fusion reporter was utilized. Cameron et al. report that deletion of *espP* or coculturing with B. thetaiotaomicron significantly increased translocation of Tir and that coculturing of Δ*espP* EHEC with B. thetaiotaomicron further increased Tir translocation. Addition of a protease inhibitor cocktail to EHEC cocultured with B. thetaiotaomicron reduced Tir translocation but did not abrogate function to the level of EHEC alone, consistent with B. thetaiotaomicron potentiating EHEC virulence by multiple mechanisms, including protein cleavage and production of succinate that induces LEE transcription (4). Finally, Cameron et al. report that levels of the T3SS needle sheath protein EspA were increased in the absence of *espP* or presence of B. thetaiotaomicron and that the presence of B. thetaiotaomicron further increased EspA filaments induced by the Δ*espP* strain. The effect of *espP* deletion suggests that EspP may limit activity of the T3SS translocon.

Further work will need to clarify the roles of EHEC and commensal proteases during infection. While evidence from Cameron et al. suggests EspP limits effector translocation and therefore virulence, both EspP and the T3SS are known virulence factors important for colonization in cows, a major reservoir for EHEC (7, 8). Future work may explore the role of EspB cleavage by EspP during EHEC infection of the colon. Additionally, testing the impact of purified EspP on infection may clarify whether deletion of *espP* has pleiotropic effects on T3SS function of EHEC. While coculturing B. thetaiotaomicron has been shown to enhance EHEC pathogenesis *in vitro* and *in vivo*, the role of the proteases during infection will likely be more fully explored. Future work may determine functional effects on infection of cleavage of EspB by testing the EspB mutants resistant to cleavage. Additional biochemical and genetic analyses may identify the relevant B. thetaiotaomicron proteases and confirm their role in infection, perhaps utilizing the murine A/E pathogen Citrobacter rodentium and reconstitution of a microbiota in germfree mice. Identification of the relevant B. thetaiotaomicron proteases will allow determination of their prevalence among Bacteroidetes and other gut commensals to determine whether variations in microbiota may affect susceptibility to EHEC and other gut pathogens that utilize the T3SS during infection. Finally, development of specific protease inhibitors could limit infection by EHEC in both humans and cattle, which has the potential to greatly reduce spread and infection by these devastating pathogens.

This work raises exciting questions about whether EHEC and other A/E pathogens evolved to use commensal proteases to regulate and mature their T3SS. In germfree mice that lack a commensal microbiota, C. rodentium LEE is dispensable, consistent with a role for the LEE-encoded T3SS in outcompeting resident microbiota to establish pathogenesis (2). While the LEE is induced on days 3 to 7 of infection in germfree mice, it is downregulated by day 12, although high pathogen burdens are maintained through day 42 (2). These findings suggest that C. rodentium may not utilize A/E in the absence of microbiota. The study by Cameron et al. suggests that A/E pathogens may rely on microbiota proteases to maximize function of the T3SS translocon essential for A/E. Future work may address the broader question of how this relationship arose and the implications for other commensal-pathogen interactions.
